# Characterization of the Whole-Genome Sequence of an H3N6 Avian Influenza Virus, Isolated from a Domestic Duck in Guangxi, Southern China

**DOI:** 10.1128/genomeA.01190-15

**Published:** 2015-10-15

**Authors:** Tingting Liu, Zhixun Xie, Sisi Luo, Liji Xie, Xianwen Deng, Zhiqing Xie, Li Huang, Jiaoling Huang, Yanfang Zhang, Tingting Zeng, Sheng Wang

**Affiliations:** a College of Life Science, Guangxi Normal University, Guilin, Guangxi Province, China; b Guangxi Key Laboratory of Animal Vaccines and Diagnostics, Guangxi Veterinary Research Institute, Nanning, Guangxi Province, China

## Abstract

A field strain of H3N6 avian influenza virus (AIV), A/duck/Guangxi/175D12/2014(H3N6), was isolated from a native duck in Guangxi Province, southern China, in 2014. All of the eight AIV gene segments were sequenced, and sequence results revealed that there were 11 amino acid deletions at the NA stalk region. The NA, PB2, and NP genes showed highest homology to H5N6 AIV, and the PA gene showed highest homology to H7N2 AIV. Phylogenetic analysis indicated that the eight AIV gene segments belonged to the Eurasian lineage. These findings provide scientific evidence of possible or potential mutations of H3N6 AIV circulating in waterfowl in southern China.

## GENOME ANNOUNCEMENT

Avian influenza viruses (AIVs) are members of the family *Orthomyxoviridae*. To date, a total of 17 hemagglutinin (HA) and 10 neuraminidase (NA) subtypes of AIV have been reported (1–3). Water fowls are carriers for most of the subtypes as their nature hosts. Domestic ducks play an important role in virus transmission from wild waterfowl species to terrestrial poultry (4). The H3 subtype AIV is most frequently isolated from feral ducks (5). Although infected domestic ducks via H3 subtype AIV do not display symptoms, they carry and shield the virus and provide an environment for the virus’s reassortment with other subtypes, such as H5 and H7 AIVs, which may cause disease in humans. Therefore, it is important to monitor H3 subtype AIV in ducks and conduct a genomic characterization to detect any potential mutation.

An H3N6 subtype AIV, A/duck/Guangxi/175D12/2014(H3N6), was isolated from an infected duck in a live poultry market in Guangxi. The eight gene segments of this duck’s H3N6 AIV were amplified by RT-PCR using AIV universal primers (6–8). The amplified PCR products were purified and cloned into the pMD-18T vector and sequenced (TaKaRa). The genomic sequence was analyzed by MEGA6.0.

The complete genomic segments include PB2, PB1, PA, NP, HA, NA, NS, and M genes, with full lengths of 2,341, 2,341, 2,233, 1,565, 1,765, 1,431, 1,027, and 890 nucleotides (nt), respectively. The NA gene lost 33 nt at positions 194 to 226. There are only six potential N-linked glycosylation sites in the NA protein (positions 51, 59, 75, 135, 190, and 391), which is different from the pigeon H3N6 AIV reported recently (9). The HA cleavage site possesses only a single basic amino acid (^340^PEKQTR↓GLF^348^), which is characteristic of low-pathogenicity AIV (10). The amino acid residues at the receptor binding site in the HA protein are Q^226^ and G^228^, which suggests that this duck H3N6 AIV would preferentially bind to alpha(2,3)-linked sialic acid receptors, which are predominant in avian species (11, 12). Analysis of potential glycosylation sites reveals that there are 6 potential N-linked glycosylation sites in the HA protein (positions 22, 38, 54, 181, 301, and 499). In addition, this duck’s H3N6 AIV possesses E and D at positions 627 and 701 of the PB2 protein, which suggests that this duck’s virus is of avian origin (13, 14).

Phylogenetic analysis indicates that all eight gene segments of this duck H3N6 AIV belongs to the Eurasian lineage. The NA gene fragment is closely related to that of H5N6 AIVs isolated in Eurasia in 2013 and 2014, as they share 98.8% to 99.3% nucleotide homology. PB2 and NP gene fragments are most closely related to A/duck/Guangdong/GD01/2014(H5N6) and A/chicken/Dongguan/3363/2013(H5N6), respectively. The PA gene fragment is most closely related to A/duck/Wenzhou/775/2013(H7N2). These data suggest that this duck’s H3N6 AIV could share similar original ancestors as those of H7N2 and H5N6 viruses in Eurasia.

In summary, this duck’s H3N6 AIV is a novel reassortant AIV, and the findings provide a better understanding of the ecology and epidemiology of H3N6 AIV circulating in southern China.

### Nucleotide sequence accession numbers.

The genome sequence of A/duck/Guangxi/175D12/2014(H3N6) was deposited in GenBank under the accession numbers KR919740 to KR919747.
